# Significant Determinants of Mouse Pain Behaviour

**DOI:** 10.1371/journal.pone.0104458

**Published:** 2014-08-07

**Authors:** Michael S. Minett, Niels Eijkelkamp, John N. Wood

**Affiliations:** 1 Molecular Nociception Group, Wolfson Institute for Biomedical Research, University College London, Gower Street, London, United Kingdom; 2 Laboratory of Neuroimmunology and Developmental Origins of Disease, University Medical Center Utrecht, Utrecht, The Netherlands; Toronto University, Canada

## Abstract

Transgenic mouse behavioural analysis has furthered our understanding of the molecular and cellular mechanisms underlying damage sensing and pain. However, it is not unusual for conflicting data on the pain phenotypes of knockout mice to be generated by reputable groups. Here we focus on some technical aspects of measuring mouse pain behaviour that are often overlooked, which may help explain discrepancies in the pain literature. We examined touch perception using von Frey hairs and mechanical pain thresholds using the Randall-Selitto test. Thermal pain thresholds were measured using the Hargreaves apparatus and a thermal place preference test. Sodium channel Nav1.7 knockout mice show a mechanical deficit in the hairy skin, but not the paw, whilst shaving the abdominal hair abolished this phenotype. Nav1.7, Nav1.8 and Nav1.9 knockout mice show deficits in noxious mechanosensation in the tail, but not the paw. TRPA1 knockout mice, however, have a loss of noxious mechanosensation in the paw but not the tail. Studies of heat and cold sensitivity also show variability depending on the intensity of the stimulus. Deleting Nav1.7, Nav1.8 or Nav1.9 in Nav1.8-positive sensory neurons attenuates responses to slow noxious heat ramps, whilst responses to fast noxious heat ramps are only reduced when Nav1.7 is lost in large diameter sensory neurons. Deleting Nav1.7 from all sensory neurons attenuates responses to noxious cooling but not extreme cold. Finally, circadian rhythms dramatically influence behavioural outcome measures such as von Frey responses, which change by 80% over the day. These observations demonstrate that fully characterising the phenotype of a transgenic mouse strain requires a range of behavioural pain models. Failure to conduct behavioural tests at different anatomical locations, stimulus intensities, and at different points in the circadian cycle may lead to a pain behavioural phenotype being misinterpreted, or missed altogether.

## Introduction

Rodent behavioural models have been important tools for furthering our understanding of the physiology underlying nociception and pain, as well as examining the pharmacological mechanisms of analgesics [1]. Several different models have been designed to assess various pain modalities, such as the Hargreaves test for noxious thermal stimuli [2] and the Randall-Selitto test for noxious mechanical stimuli [3]. From the mid-1990s, application of these behavioural assays to transgenic mice has increased our understanding of the molecular and cellular mechanisms underlying nociception and pain. Recently, cell ablation studies utilising the Cre-loxP system [4], [5] have demonstrated that distinct sensory subpopulations underlie distinct pain modalities, distinguishing mechanical and thermal pain [6], [7].

Many transgenic studies use a seemingly standardised array of mouse behavioural pain assays. Comparing the results of these behavioural pain assays can produce contradictory findings. For example, Kwan *et al.* produced a TRPA1-knockout mouse strain, which lack the S5 and S6 transmembrane domains and the pore-loop that contains the channel's selectivity filter (encoded by exons 22, 23 & 24). Kwan *et al.* assessed touch sensitivity by probing the plantar surface of the hindpaw with calibrated von Frey filaments, using the ‘repeated measures’ paradigm, with withdrawal thresholds determined as two positive responses out of eight von Frey hair applications [8]. They showed a trend that these TRPA1-knockout mice have higher withdrawal thresholds, as well as significantly reduced responses to suprathreshold von Frey stimuli [9]. Petrus *et al.* assessed the mechanical sensitivity of the same strain of TRPA1-knockout mice using a Dynamic Plantar Aesthesiometer (an automatic von Frey machine), with an increasing force paradigm [8]. This method produced a much less pronounced phenotype, although the authors highlighted that the use of different instrumentation may account for this difference [10]. In contrast, Bautista *et al.* produced a separate TRPA1-knockout mouse strain by deleting the pore-loop only (encoded by exon 23), which showed no significant difference in response to the von Frey test using the ‘up and down’ [8], [11] paradigm [12]. More recently, Andersson *et al.* assessed mechanical sensitivity of the Kwan TRPA1-knockout strain using an Analgesymeter (Randall-Selitto test), which applies a constant increasing noxious pressure stimulus to the dorsal surface of the hind paw using a blunt conical probe. The Randall-Selitto test showed significantly higher thresholds in TRPA1-knockout mice compared to wildtype littermates [13]. At first glance these studies seem contradictory, as Kwan *et al.* and Andersson *et al.* conclude that TRPA1 contributes to acute mechanical nociception whilst Bautista *et al.* and Petrus *et al.* state that it does not. In combination, however, these studies suggest the TRPA1 has a role in suprathreshold but not threshold behavioural responses to mechanical stimuli applied to the hindpaw.

Different mouse strains have been shown to display variable sensitivity to pain in behavioural assays [14], [15]. Similarly behavioural state has a role; for example grooming can result in hypoalgesia [16]. Other factors, such as experimenter identity, animal handling and testing order, and environmental factors, such as cage density, time of day and humidity, have been shown to influence pain sensitivity in mouse behavioural models [17]. Circadian rhythms have also been shown to influence pain perception both in experimental and clinical studies [18]; diurnal rhythms for heat pain were described more than 30 years ago [19]. Here we demonstrate that other factors, particularly the intensity and location of painful stimuli are also important for uncovering the exact role of a candidate gene or neuronal subpopulation in nociception and pain.

## Results

### Mechanosensory responses

**Figure 1:**
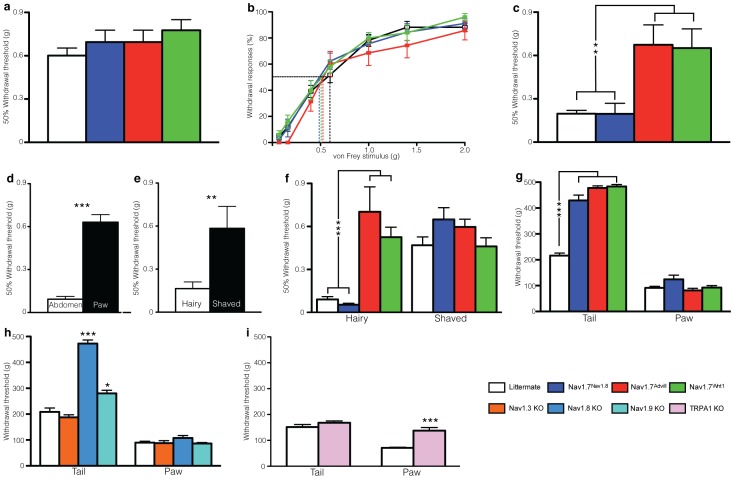
Comparison of different transgenic mice reveals test-site and stimulus-intensity specific mechanosensory responses. Nav1.7^Nav1.8^ mice (blue columns, n = 7), Nav1.7^Advill^ mice (red column, n = 9) and Nav1.7^Wnt1^ mice (green column, n = 9) mice show normal responses to von Frey hairs applied using either the up-down method (**a**) or the repeated measures method in comparison to littermate mice (white columns, n = 36). (**b**). Both Nav1.7^Advill^ mice (n = 9) and Nav1.7^Wnt1^ mice (n = 9) show a behavioural deficit in response to the abdominal von Frey test in comparison to Nav1.7^Nav1.8^ mice (n = 7) and littermate mice (n = 36) (**c**). The abdomens of C57BL/6 (n = 12) mice are significantly more sensitive than the plantar surface of the hindpaw (**d**), which is loss if the abdomen is shaved (**e**). Shaving the abdominal hair attenuates the sensitivity to von Frey hair stimulation of Nav1.7^Nav1.8^ (n = 10) and littermate mice (n = 21) but has no effect of Nav1.7^Advill^ (n = 7) or Nav1.7^Wnt1^ mice (n = 11) (**f**). Nav1.7^Nav1.8^ (n = 14), Nav1.7^Advill^ (n = 8) Nav1.7^Wnt1^ (n = 9) show a significant increase withdrawal threshold in response to the Randall-Siletto test when applied to the tail but not the paw when compared to littermate (n = 26) mice (**g**). Nav1.8KO (light blue column, n = 11) and Nav1.9KO (turquoise column, n = 8) but not Nav1.3KO (yellow column, n = 6) show a significant increase withdrawal threshold in response to the Randall-Siletto test when applied to the tail when compared to littermate (n = 27) mice, however no difference is seen when applied to the paw (**h**). TRPA1 KO mice (pink columns, n = 8) show a behavioural deficit to Randall-Selitto test applied to the paw but not tail in comparison to littermate mice (white columns, n = 8) (**i**). Data analysed by two-way analysis of variance followed by a Bonferroni post-hoc test. Results are presented as mean ± S.E.M. ** *P*<0.01 and *** *P*<0.001 (individual points).

We assessed mechanosensation at a number of anatomical locations with sodium channel knockout transgenic mouse strains, and found distinct mechanisms at play in the hindpaw, tail and hairy skin of the abdomen (Figure 1). *Floxed* (*Scn9a*) Nav1.7 mice were crossed with different tissue-restricted Cre mouse strains to make; a nociceptor-specific (Nav1.7^Nav1.8^), a pan-sensory neuron (Nav1.7^Advill^) and a pan-sensory and sympathetic neuron (Nav1.7^Wnt1^) knockout mouse strain [20]. Deleting Nav1.7 within these different sets of peripheral sensory neurons did not alter behavioural responses to von Frey hairs applied to the glabrous skin of the hindpaw plantar surface using the ‘up and down’ method (Figure 1a) or ‘repeated measures’ method (Figure 1b). In contrast, behavioural deficits are seen in the Nav1.7^Advill^ and Nav1.7^Wnt1^ mice but not in the Nav1.7^Nav1.8^ mice when the same von Frey hairs were applied to the hairy skin of the abdomen (Figure 1c). The hairy skin of the abdomen is thus more sensitive than the glabrous skin of the hindpaw (Figure 1d), but removing the hair from the abdomen of C57BL/6 mice raises the 50% response threshold to that of the hindpaw (Figure 1e). The reduction in mechanical sensitivity is still present up to 48 hours after the hair on the abdomen has been removed. After removing the abdominal hair of Nav1.7^Nav1.8^ mice, the 50% response threshold was similar to littermate controls, while abdominal hair removal did not affect the increased 50% mechanical threshold of either Nav1.7^Advill^ or Nav1.7^Wnt1^ mice compared to littermates controls (Figure 1f).

Behavioural responses to the Randall-Selitto test also vary depending upon body location. The threshold of wild type tail responses to noxious mechanical stimulation is lower than that measured at the hindpaw. As with von Frey hair responses, this may reflect the differential composition of sensory neurons innervating the tissues of these two different body locations, such as in glabrous and hairy skin [21]. Figure 1e shows increased response thresholds for all three Nav1.7 knockout strains when the Randall-Selitto test is applied to the tail, but not the paw (Figure 1g). This body-location specific increased response threshold to the Randall-Selitto test applied to the tail but not the paw was also seen in Nav1.8-knockout (KO), as well as Nav1.9-KO mice, but not in Nav1.3-KO mice (Figure 1h). In contrast, TRPA1-knockout mice show a behavioural deficit when the Randall-Selitto test is applied to the paw [13], but not the tail (Figure 1i).

**Figure 2:**
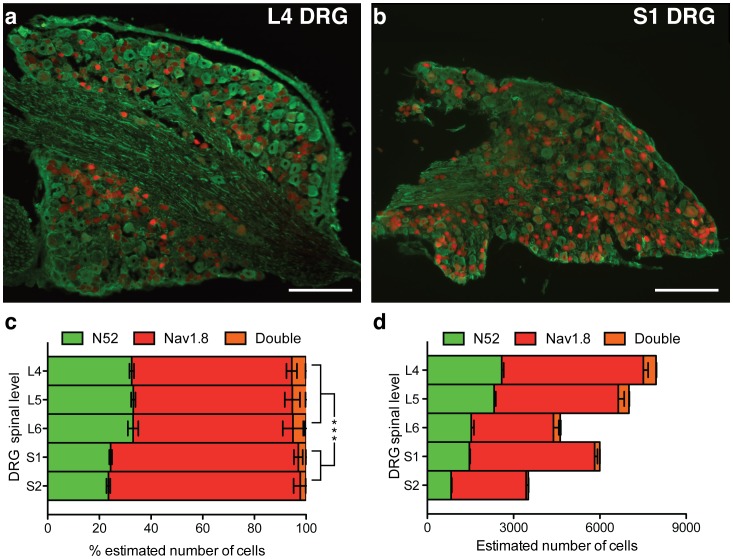
The DRG innervating the hindpaw and tail consist of different ratios of neuronal subpopulations. Example section of an L4 (**a**) and an S1 (**b**) DRG (N52: green, Nav1.8: red, scale bar = 250 µm). Overall percentage of estimated number of N52, Nav1.8 and double stained cells within L4 (n = 52), L5 (n = 43), L6 (n = 32), S1 (n = 18) and S2 (n = 17) DRG (**c**). Total estimated number of N52, Nav1.8 and double-stained cells within L4 (n = 52), L5 (n = 43), L6 (n = 32), S1 (n = 18) and S2 (n = 17) DRG (**d**). All data analysed by two-way analysis of variance followed by a Bonferroni post-hoc test. Results are presented as mean ± S.E.M. ** P<0.01 and *** P<0.001 (individual points).

To get an insight into the presence of Nav1.8-positive sensory neurons in the DRG that innervate specific anatomical regions, we crossed mice expressing Nav1.8-Cre with mice expressing a floxed-stop tdTomato fluorescent protein (Nav1.8^Tomato^) so that all Nav1.8-positive neurons are labelled [22]. Example sections of dorsal root ganglia (DRG) from Nav1.8^Tomato^ mice at the 4^th^ lumbar spinal level, which innervate the hindpaw (L4 - Figure 2a) contain proportionally less Nav1.8-positive sensory neurons than DRG at the 1^st^ sacral spinal level, which innervate the tail [23] (S1 - Figure 2b). DRG at spinal levels L4, L5 & L6, innervating the hindpaws consists of ∼61% Nav1.8-postive, ∼33% neurofilament-positive and ∼6% double-stained DRG neurons, whereas DRG at spinal levels S1 and S2, innervating the tail consist of ∼72% Nav1.8-postive, ∼24% neurofilament-positive and ∼4% double-stained DRG neurons (Figure 2c). The total number of DRG neurons found at different spinal level also varies dramatically (Figure 2d). These differences in total cell number and relative proportions may contribute to the behavioural difference seen in the Randall-Selitto test, although this does not prove a causal link.

### Distinct stimulus-intensity specific responses to noxious heat

**Figure 3:**
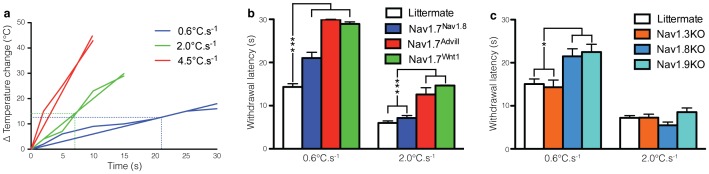
Comparison of different transgenic mice reveals stimulus-intensity specific responses to noxious thermal stimuli. Behavioural responses of different Nav1.7 tissue-specific knockouts to the Hargreaves test applied to the hindpaw. (**a**) Nav1.7^Nav1.8^ mice (blue columns, n = 14), Nav1.7^Advill^ mice (red column, n = 7) and Nav1.7^Wnt1^ mice (green column, n = 12) all show a behavioural deficit in response to the Hargreaves test at a heat ramp of 0.6°C.s^−1^ in comparison to littermate mice (white columns, n = 27), however only Nav1.7^Advill^ and Nav1.7^Wnt1^ mice show a behavioural deficit in response to the Hargreaves test at a heat ramp of 2.0°C.s^−1^. (**b**) Nav1.8KO mice (light blue column, n = 6) and Nav1.9KO mice (turquoise column, n = 10) but not Nav1.3KO mice (orange column, n = 6) show a significantly increased withdrawal latency to the Hargreaves test at a heat ramp of 0.6°C.s^−1^ in comparison to littermate mice (white columns, n = 18), however this significant increase is lost the when the Hargreaves test is conducted using a heat ramp of 2.0°C.s^−1^. Data analysed by two-way analysis of variance followed by a Bonferroni post-hoc test. Results are presented as mean ± S.E.M. * *P*<0.05 and *** *P*<0.001 (individual points).

Figure 3 shows that different stimulus intensities of the same pain modality and test location require distinct neuronal subpopulations. Figure 3a shows that changing the light intensity of the Hargreaves' apparatus results in different heat ramp. A heat ramp of 0.6°C.s^−1^ applied to the plantar surface of the hindpaw reveals a significant increased response threshold for Nav1.7^Nav1.8^, Nav1.7^Advill^ and Nav1.7^Wnt1^, when compared to littermate controls. However, applying a heat ramp of 2.0°C.s^−1^ to the plantar surface of the hindpaw shows that only Nav1.7^Advill^ and Nav1.7^Wnt1^ mice display a behavioural deficit (Figure 3b). Similarly, Nav1.8KO and Nav1.9KO mice show behavioural deficits in response to a heat ramp of 0.6°C.s^−1^ but not 2.0°C.s^−1^ (Figure 3c). Interestingly both the 0.6°C.s^−1^ and 2.0°C.s^−1^ heat ramp trigger a withdrawal response following a temperature rise of ∼13°C (figure 3a). Finally, Nav1.3KO show normal behavioural responses to both a 0.6°C.s^−1^ and 2.0°C.s^−1^, heat ramp, suggesting that Nav1.3 is not required for any reflex responses to noxious thermal stimuli (Figure 3c). These data suggest that Nav1.8-positive DRG neurons are have a non-redundant role in mediating slowly transduced responses to the 0.6°C.s^−1^ heat ramp but not responses to the 2.0°C.s^−1^ heat ramp. It also suggests Nav1.8-negative DRG neurons mediate the response to the 2.0°C.s^−1^ heat ramp, although this response may also require input from Nav1.8-positive DRG neurons.

### Distinct stimulus-intensity specific responses to cooling and noxious cold

**Figure 4:**
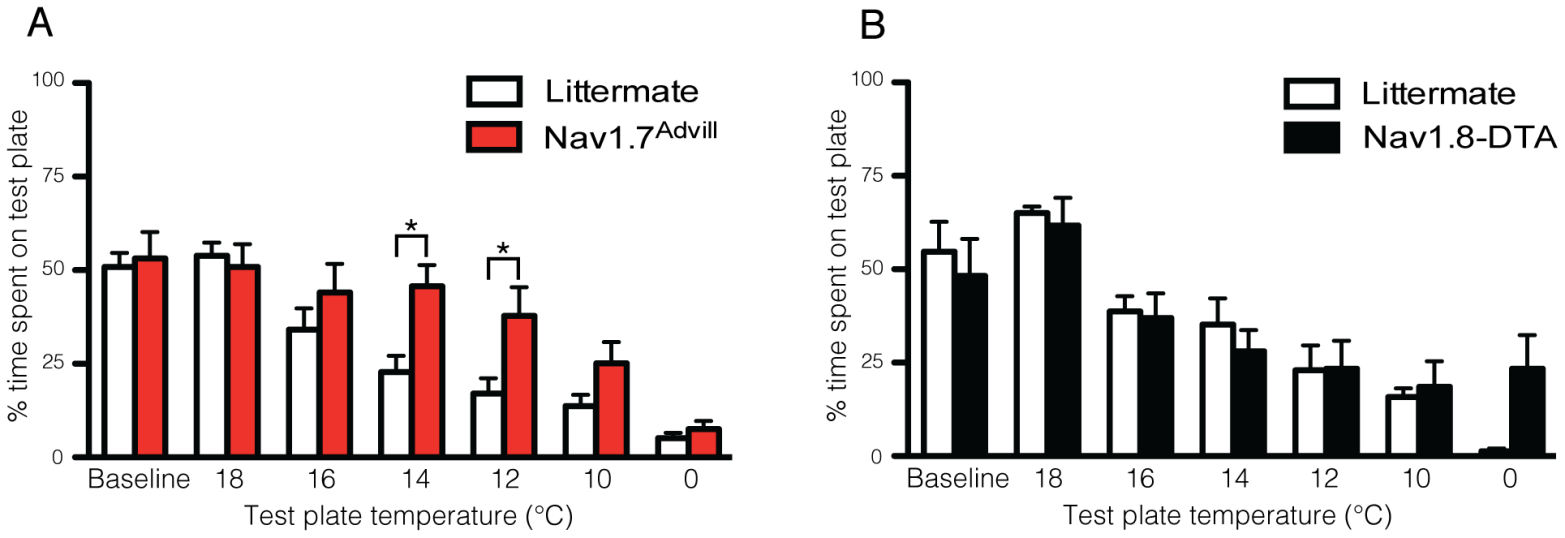
Comparison of different transgenic mice reveals distinct mechanisms underlie responses to cooling and noxious cold stimuli. (**a**) Nav1.7^Advill^ mice (red columns, n = 8) avoid the 0°C test plate to the same extent as littermate controls (white columns, n = 8) in the thermal place preference test but show a behavioural deficit in the noxious cooling range (14–12°C). (**b**) Nav1.8-DTA mice (black columns, n = 6) avoid the cooling stimuli to the same extent as littermate controls (white columns, n = 6) in the thermal place preference test but show a trend indicating a behavioural deficit in response to 0°C. Data analysed by two-way analysis of variance followed by a Bonferroni post-hoc test. Results are presented as mean ± S.E.M. * *P*<0.05 (individual points).

Figure 4a shows the response of Nav1.7^Advill^ mice to a dynamic thermal place preference (TPP) behavioural assay. Nav1.7^Advill^ mice show an attenuated response to cooling stimuli (14 & 16°C) but not to ‘extreme cold’ (0°C). In contrast, figure 4b shows that mice where all Nav1.8-positive neurons have been transgenically ablated using Diphtheria toxin (Nav1.8-DTA) [6] show normal responses to cooling stimuli but an attenuated response to ‘extreme cold’. As with responses to noxious heat stimuli these data indicate that a range of thermal stimuli in needed in order to interpretation thermal responses as the mechanism underpinning responses to different temperatures ranges differs.

### Circadian rhythms and pain

**Figure 5:**
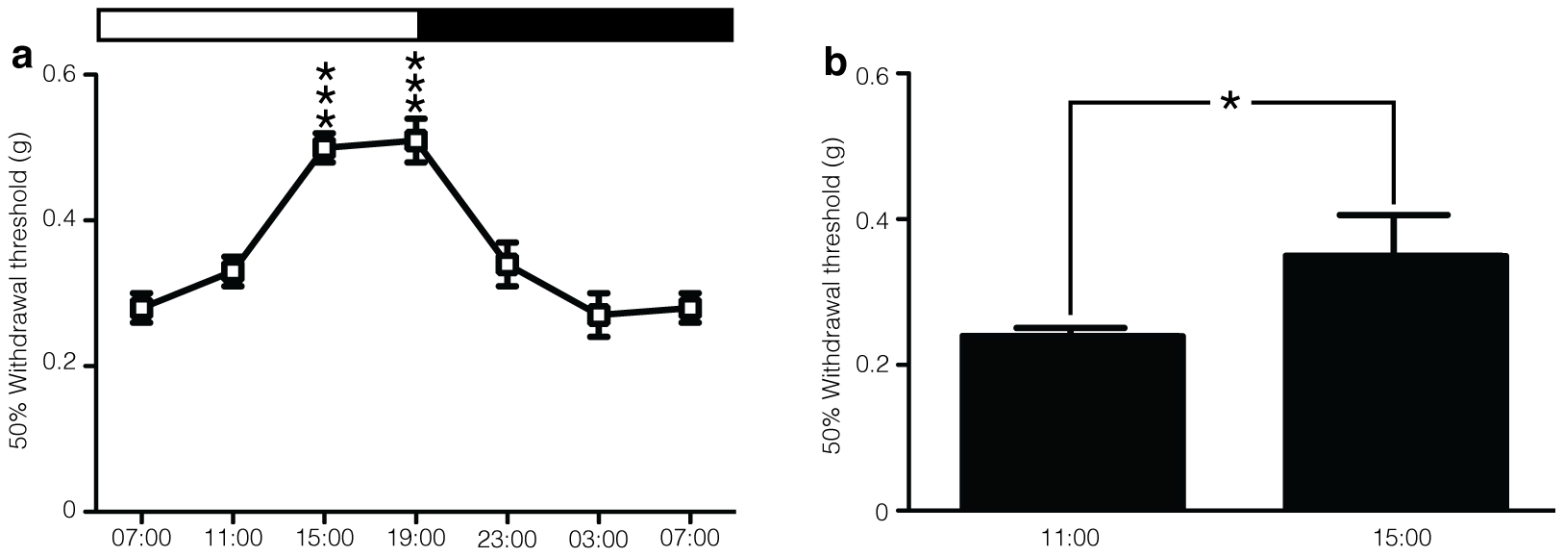
The affect of circadian rhythm on von Frey responses over a 24-hour period. (**a**) Behavioural responses of C57BL/6 mice to the von Frey hairs applied to the hindpaw over a 24 h period. Measurements were taken every 4 hours starting at 07:00. (**b**) Behavioural responses of Nav1.8-DTA mice to the von Frey hairs applied to the hindpaw over a 24 h period. (**a**) Data analysed by two-way analysis of variance followed by a Bonferroni post-hoc test and (**b**) t-test. Results are presented as mean ± S.E.M. * *P*<0.05, *** *P*<0.001.

To investigate the influence of circadian rhythm on the outcome measures of mouse behavioural pain assays we measured responses to von Frey hairs applied to the plantar surface of the hindpaw every 4 hours over a 24-hour period. The 50% withdrawal threshold to von Frey hair stimulation significantly increased during the light (inactive) period, peaking between 15:00 and 19:00 and decreased during the dark (active) phase with the lowest threshold observed between 03:00 and 07:00 (figure 5a). These circadian changes do not require Nav1.8-positive nociceptors since Nav1.8-DTA mice show a similar circadian rhythm in their response to light touch (figure 5b).

## Discussion

Pain research using behavioural models in transgenic mice is a continuing necessity for furthering our understanding of the fundamental mechanisms of pain, as well as identifying novel analgesics. Here we show that behavioural responses to the same sensory stimulus at different anatomical locations involve distinct underlying mechanisms and neuronal subpopulations. Mammalian skin can be divided into two major types: 1) Glabrous (non-hairy), which contains four mechanosensory end organs: Pacinian corpuscles, Ruffini endings, Meissner corpuscles, and Merkel's discs. 2) Hairy skin, which comprises three major hair types: zigzag, awl/auchene, and guard that act as specialized mechanosensory organs [21]. Additionally, free nerve endings are found in the epidermis of both glabrous and hairy skin. Different neuronal subtypes and peripheral innervation patterns suggests that glabrous and hairy skin represent morphologically distinct, but highly specialized, mechanosensory organs, each capable of mediating unique functional responses or aspects of touch (see Abraira and Ginty, 2013 [21] for more detailed discussion). Deleting Nav1.7 in peripheral sensory neurons does not alter touch sensation in the hindpaw, but attenuates sensitivity to touch in hairy skin. This demonstrates that mechanosensory properties of hair follicles are Nav1.7-dependent. Recent work by Li *et al.* demonstrated that each of the different hair follicle types present in mice are functionally distinct mechanosensory end organs that are differentially innervated by unique and invariant combinations of Aβ, Aδ and C-fibres [24]. Shields *et al.* showed, however, that Nav1.8-Cre is only expressed in a subpopulation of sensory neurons that innervate some types of hair follicles [25], which may be required for detection of social stroking/grooming [24]. In contrast, Nav1.8-negative sensory neurons innervate the hair follicles that may detect mechanical stimuli, such as light contact with foliage during normal rodent locomotion [24]. This reflects the fact that responses to von Frey hairs applied to the abdomen are only altered when Nav1.7 is deleted within the Nav1.8-negative (in addition to the Nav1.8-positive) population of sensory neurons. Following removal of the hair follicles, the free nerve endings found in the epidermis of both the glabrous and hairy skin may play more prominent roles in responses to von Frey hairs applied to the abdomen [21].

As with responses to von Frey hairs, behavioural responses to the Randall-Selitto test vary depending upon anatomical location. Disrupting the function of Nav1.8-positive DRG neurons, which seem to form a greater proportion of the sensory neurons innervating the tail, increases the response threshold of the tail, but not the response threshold of the hind-paws in the Randall-Selitto test. In contrast, TRPA1 is required for lower threshold Randall-Selitto responses of the hindpaw but not the higher-threshold Randall-Selitto responses of the tail. It should be noted that the low-threshold Randall-Selitto response of the hindpaw is not related to von Frey test responses. TRPA1-knockout mice show normal von Frey thresholds [10], [12] but are less sensitive to suprathreshold von Frey stimuli applied to the hindpaws [9].

In addition to the anatomical location, detection of different intensities of the same stimulus involve distinct underlying mechanisms and neuronal subpopulations.

Previous findings showed that spinal and supraspinal heat processing is different in Nav1.7^Advill^ and Nav1.7^Wnt1^ mice, where only Nav1.7^Wnt1^ mice show a deficit in response to the hot-plate test [20]. Together this demonstrates the existence of at least three distinct subpopulations of peripheral neurons that contribute to ‘heat pain’ in mice; 1) Nav1.8-positive sensory neurons that contribute to the 0.6°C.s^−1^ Hargreaves' test responses, 2) Nav1.8-negative sensory neurons that contribute to the 2°C.s^−1^ Hargreaves' test responses, and 3) sympathetic neurons, in concert with sensory neurons contribute to supraspinally mediated hot-plate responses [20].

Similar to heat pain, distinct mechanisms underlie cold pain in mice. Previously, Abrahamsen et al. showed that Nav1.8-positive DRG neurons are critical for behavioural responses to 0°C [6]. More specifically, Zimmermann *et al.* have shown that Nav1.8, but not Nav1.7 [20], is essential for behavioural responses below 10°C, specifically ‘extreme cold’ 0°C or below. Peier *et al.* showed that TRPM8 is activated at a temperature threshold of ∼28°C, with currents increasing in magnitude as the temperature decreases down to 8°C [26]. Thus TRPM8 activity spans the range from innocuous cooling down towards noxious cold temperatures. TheTRPM8 knockout mouse strain, shows an attenuated response to cooling stimuli between ∼28°C and ∼8°C, but not ‘extreme cold’ below 0°C in the TPP test [27], [28]. Nav1.7^Advill^ mice show similar response to the TPP test, where avoidance of cooling stimuli between ∼14°C and ∼12°C, but not ‘extreme cold’ is blunted (Figure 4a). In contrast, figure 4b shows that Nav1.8-DTA mice show normal responses to cooling stimuli but an attenuated response to ‘extreme cold’. Application of acetone to the skin leads to a rapid temperature decrease spanning the cooling range [8]. Previously, a behavioural deficit has been shown in Nav1.7^Advill^ but not Nav1.7^Nav1.8^ mice in responses to application of acetone to the plantar surface of the hind-paw [20], [29]. This demonstrates that Nav1.8-negative sensory neurons are required for behavioural responses to a cooling acetone stimulus. Comparing the behavioural responses of Nav1.7 knockout mice [20], and transgenic mice lacking Nav1.8-positive neurons [6] in the TPP test (figure 4b) shows that Nav1.8-positive neurons are required for the detection of ‘extreme cold’ but not cooling stimuli. As with mechanosensation, these data on thermal pain processing demonstrate how important information about the contribution of a candidate gene or compound to ‘thermal pain’ can be misinterpreted if the full range of thermal pain tests (i.e. Hargreaves', hot-plate, acetone and thermal place preference tests) is not examined.

Circadian rhythm can also alter responses to light touch and this does not require Nav1.8-positive nociceptors. Circadian variation has some implication for testing analgesics Kusunose *et al.* showed that the efficacy of gabapentin in attenuating mechanical allodynia in the Seltzer neuropathic pain model [30] was subject to a circadian rhythm [31]. Thus consistent timing of experiments is an important factor to consider when designing pain phenotyping experiments [17].

## Conclusions

The data presented here demonstrate that the role of a candidate gene or analgesic compound can be misinterpreted or even missed, if only limited behavioural assays are conducted. These intricacies of phenotyping may also help explain seemly contradictory finding from different groups, as subtle differences in experimental approach can lead to different results.

## Methods

### Animals

All experiments were performed with prior approval from the UK Home Office under a Home Office project license (PPL 70/7382). Experiments were conducted using both male and female wildtype littermate and knockout mice, all of which were at least 6 weeks old when tested. Observers who performed behavioural experiments were blind to the genotype of the animals. The production of the following transgenic mice was documented, respectively, in following articles; Nav1.8-Cre mice [32], Advillin-Cre mice [20], [33], Wnt1-Cre mice [34], Nav1.8^Tomato^ mice [22], Nav1.3 global knockout mice [35], floxed Nav1.7 mice [36], Nav1.8 global knockout mice [37], Nav1.9 global knockout mice [38] and Nav1.8-DTA mice [6].

### Generation of Nav1.7 conditional knockout mouse strains

We used the Cre-loxP system to generate a number of conditional Nav1.7 knockout mouse strains. Floxed (*SCN9A*) Nav1.7 mice were crossed with strains where Cre expression is driven by either the Nav1.8 promoter (Nav1.7^Nav1.8^), expressed in >90% of neurons expressing markers of nociceptors [25], [32], the Advillin promoter (Nav1.7^Advill^), expressed in all DRG neurons [20], and the Wnt1 promoter (Nav1.7^Wnt1^), expressed in tissue derived from the neural tube, including sensory and sympathetic neurons [34]. Additionally, a nociceptor labelled strain (Nav1.8^Tomato^) was generated by using Nav1.8-Cre to remove the loxP-flanked Stop cassette preventing the expression of exceptionally bright red fluorescent protein tdTomato [22]. Similarly, a nociceptor-ablated mouse strain (Nav1.8-DTA) was generated using Nav1.8-Cre to remove the loxP-flanked stop cassette preventing the expression of Diphtheria Toxin A (DTA)-subunit [6].

### Behavioural assays

All behavioural experiments were performed between 12:00 and 15:00, unless stated otherwise. Mechanical nociceptive thresholds were measured using modified version of the Randall-Selitto test that applies pressure to the tail via a 3 mm^2^ blunt conical probe [3], [8] with a 500 gram cut-off. Alternatively, the probe was applied to the dorsal surface of the hindpaws [13], [8], with a 250 gram cut-off. Touch perception was measured using the up-down method for obtaining the 50% threshold using von Frey hairs as described by [11], [8]. The access touch perception in hair and non-hair skin von Frey hairs were applied to the plantar surface of the hindpaw or the inferior half of the abdomen, respectively. Abdominal hair was removed using hair clippers (Wella, UK).

Thermal nociceptive thresholds were determined by measuring paw-withdrawal latency using the Hargreaves apparatus [2], [8]. As well as the hot-plate test (50 & 55°C) [39]. A thermal place preference (BioSeb) was used to assess cold avoidance [20]. Mice were placed in a plexiglas chamber with two adjacent thermal surfaces both with an accuracy of ±0.1°C. Mouse movements were recorded with a video tracking system during a 2-minute test period. During which one plate was kept at a constant temperature whilst the other plate was set to test temperatures, the plate temperatures were then reversed. An average of the two 2-minute test periods was recorded.

### Immunocytochemistry

DRGs were excised from animals perfused with 4% PFA. Serial 10 µm sections were collected. Slides were washed and blocked in 10% goat serum in PBS +0.3% Triton for 1 hour at room temperature and incubated in the primary antibody overnight at 4°C. Primary antibodies were detected by incubating with the secondary antibody at room temperature for 2 hours.

### Cell counting

Tissue samples were visualised using a Leica DMRB microscope, a Hamamatsu ORCA-R2 digital camera and HCIamge 2.0.1.16 software. The sample images were analysed using the cell counter plugin for ImageJ 1.47a. The number of cells per DRG was estimated by averaging cell-counts from three animals. For each animal ∼15 section images (each separated by ∼30 µm) were counted.

### Statistics

Data were analysed using the GraphPad Prism 5. Student's t-test (two-tailed) was used for comparison of difference between two groups. Multiple groups were compared using one-way or two-way analysis of variance with a Bonferroni post-hoc test.
