# Correction: Significant Determinants of Mouse Pain Behaviour

**DOI:** 10.1371/journal.pone.0245813

**Published:** 2021-01-15

**Authors:** Michael S. Minett, Niels Eijkelkamp, John N. Wood

There are a number of errors in the caption for Fig 3, “Comparison of different transgenic mice reveals stimulus-intensity specific responses to noxious thermal stimuli.” Please see the complete, correct Fig 3 caption here.

**Fig 3:**
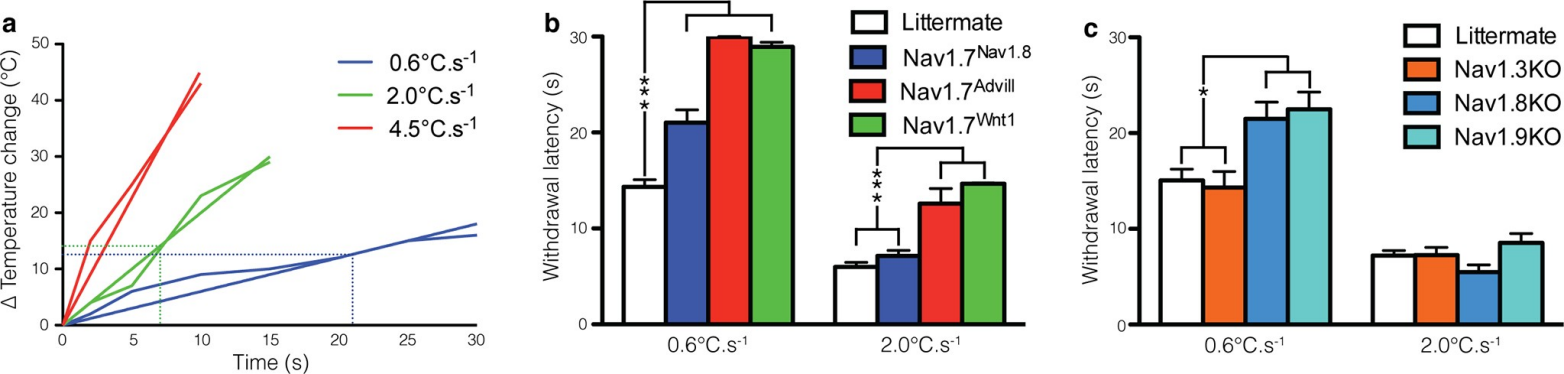
Comparison of different transgenic mice reveals stimulus-intensity specific responses to noxious thermal stimuli. Behavioural responses of different Nav1.7 tissue-specific knockouts to the Hargreaves test applied to the hindpaw. (**a**) Nav1.7^Nav1.8^ mice (blue columns, n  =  14), Nav1.7^Advill^ mice (red column, n  =  7) and Nav1.7^Wnt1^ mice (green column, n  =  12) all show a behavioural deficit in response to the Hargreaves test at a heat ramp of 0.6°C.s^−1^ in comparison to littermate mice (white columns, n  =  27), however (**b**) only Nav1.7^Advill^ and Nav1.7^Wnt1^ mice show a behavioural deficit in response to the Hargreaves test at a heat ramp of 2.0°C.s^−1^. (**c**) Nav1.8KO mice (light blue column, n  =  6) and Nav1.9KO mice (turquoise column, n  =  10) but not Nav1.3KO mice (orange column, n  =  6) show a significantly increased withdrawal latency to the Hargreaves test at a heat ramp of 0.6°C.s^−1^ in comparison to littermate mice (white columns, n  =  18), however this significant increase is lost the when the Hargreaves test is conducted using a heat ramp of 2.0°C.s^−1^. Data analysed by two-way analysis of variance followed by a Bonferroni post-hoc test. Results are presented as mean ± S.E.M. * *P*<0.05 and *** *P*<0.001 (individual points).
